# Winter Storm Uri, Mortality, and Health Care Use of Nursing Home Residents

**DOI:** 10.1001/jamanetworkopen.2025.4111

**Published:** 2025-04-08

**Authors:** Brian Downer, Alexandra Holland, Shuang Li, Huiwen Xu

**Affiliations:** 1 The University of Texas Medical Branch, School of Public and Population Health, Galveston; 2 Sealy Center on Aging, The University of Texas Medical Branch, Galveston; 3 Nell Hodgson Woodruff School of Nursing, Emory University, Atlanta, Georgia

## Abstract

**Importance:**

Extreme weather events disproportionately affect vulnerable populations, particularly nursing home residents. In February 2021, Winter Storm Uri caused unprecedented power outages and water shortages across Texas. However, its impact on nursing home residents remains poorly understood.

**Objective:**

To assess the association of power outages and water shortages during Winter Storm Uri with mortality and health care utilization among long-stay nursing home residents in Texas.

**Design, Setting, and Participants:**

A retrospective cohort study of long-stay nursing home residents before and after Winter Storm Uri (February 14 to February 17, 2021) in Texas. Data analysis was conducted from March 2024 to January 2025.

**Exposure:**

Residing in a nursing home that experienced a power outage or water shortage during Winter Storm Uri.

**Main Outcomes and Measures:**

The outcome measures were weekly rates of mortality, clinician visits to nursing homes, emergency department (ED) visits, and hospitalizations among long-stay nursing home residents. Medicare claims were used to determine weekly mortality, clinician visits, ED visits, and hospitalizations 6 weeks before and 6 weeks after the storm. Differences-in-differences (DiD) analysis using 2-level logistic regression models estimated changes in outcomes associated with nursing home power or water loss.

**Results:**

Of 1174 nursing homes, 231 (19.7%) reported power or water outages. The sample included 45 439 long-stay residents, with 8521 in facilities with outages (mean [SD] age, 80.07 [12.21] years; 5664 female [66.47%]) and 36 918 in facilities without outages (mean [SD] age, 80.42 [11.92] years; 12 705 female [65.59%]). DiD analysis found that residents of nursing homes with outages had a 0.13% (95% CI, 0.03% to 0.23%) higher mortality rate in the 3 to 5 weeks after the storm than those without outages. There were no significant differences in clinician visits (DiD, −0.31%; 95% CI, −1.08% to 0.47%), ED visits (DiD, 0.11%; 95% CI, −0.12% to 0.34%), or hospitalizations (DiD, 0.07%; 95% CI, −0.10% to 0.23%).

**Conclusions and Relevance:**

In this cohort study of nursing home residents in Texas, power and water outages during Winter Storm Uri were associated with increased mortality but not ED visits or hospitalizations, highlighting the vulnerability of nursing home residents during extreme weather events.

## Key Points

**Question:**

Were power and water outages during Winter Storm Uri associated with mortality and health care utilization of long-stay nursing residents in Texas?

**Findings:**

In this cohort study of 45 439 long-stay nursing residents in 1174 nursing homes, weekly mortality was significantly higher for long-stay residents of nursing homes that had a power or water outage than nursing homes that did not. Health care utilization was similar between both groups.

**Meaning:**

These findings highlight the vulnerability of nursing home residents during extreme weather events.

## Teaser

This cohort study assesses associations of Winter Storm Uri with mortality and health care utilization among long-stay nursing home residents in Texas.

## Introduction

Climate change has increased the frequency and intensity of major weather events. The repeated occurrence of major weather events in specific regions of the country can provide people with a sense of familiarity and preparedness, but major weather events are more frequently occurring in regions where people do not expect them.^1,2^ A notable example is Winter Storm Uri, which occurred in Texas and other parts of the US in February 2021.^3,4^ Over a 5-day period, Texas experienced record-breaking cold temperatures, snow, and ice that strained the Texas power grid. On February 14, 2021, the Electric Reliability Council of Texas used rolling blackouts to manage the high demand for electricity, leaving millions of people without reliable power for several days.^5,6^ The power outages and freezing temperatures caused a severe water crisis as frozen pipes burst, and 14 million people were under boil water notices.^7^

It is well known that major weather events disproportionately affect older adults.^8,9,10^ Nursing home residents are especially vulnerable because they often have physical and cognitive limitations, multiple chronic conditions, and rely on staff to meet their daily care needs.^11,12^ Most research on the associations of major weather events with nursing home residents has focused on hurricanes.^13,14,15^ Nursing home residents are at an increased risk for poor outcomes, including hospitalizations and death, for months after a hurricane has passed.^16,17^

The widespread effects of Winter Storm Uri made it an unprecedented event in Texas. The Texas Department of State Health Services attributed 246 deaths to Winter Storm Uri, most of which (58.8%) were among adults aged 60 years and older.^18^ Another government report^19^ found that 578 of 1212 nursing homes reported incidents during Winter Storm Uri, including 118 facilities with blackouts or no electricity and 111 facilities with burst pipes, water shortages, or no water. These variations provide an opportunity to assess the associations of Winter Storm Uri with nursing home residents.

In this study, we investigated differences in mortality and health care utilization among Texas nursing home residents according to whether a nursing home reported losing power or water during Winter Storm Uri. We hypothesized that loss of power or water would be associated with fewer clinician visits to nursing homes but higher mortality, as well as more emergency department (ED) visits and hospitalizations in the 6 weeks after Winter Storm Uri.

## Methods

### Data and Study Sample

This cohort study was approved by the University of Texas Medical Branch institutional review board with a waiver of informed consent because the study was a secondary analysis of deidentified data, in accordance with 45 CFR §46. This study followed the Strengthening the Reporting of Observational Studies in Epidemiology (STROBE) reporting guideline.^20^ The Texas Health and Human Services Commission provided information on whether nursing homes lost power or water during Winter Storm Uri via a statewide survey.^19^ We used the Minimum Data Set data to identify long-stay residents who resided in Texas nursing homes during the storm. Residents’ characteristics for sex, age, race and ethnicity, Medicaid status, and date of death date were from the Medicare Beneficiary Summary File in the second quarter of 2021. Race and ethnicity categories included Black, Hispanic, non-Hispanic White, and other race or ethnicity (defined as American Indian or Alaska Native, Asian or Pacific Islander, and any race or ethnicity not otherwise specified); race and ethnicity were included to account for known racial and ethnic disparities in the quality and outcomes of nursing home care. The Centers for Medicaid and Medicare Services (CMS) uses an algorithm for race and ethnicity based on a Medicare beneficiary’s name, geographic location, and the language a beneficiary prefers CMS to use when sending information.^21^ Information for nursing home characteristics (the number of beds in a facility, whether the facility was in a rural or urban area, and ownership status [nonprofit, government, or profit]) was from the Provider of Services data. We used both Medicare fee-for-service claims and Medicare Advantage encounter data (inpatient, outpatient, and carrier) to identify health care utilization during the 6 weeks before, the week of, and the 6 weeks after Winter Storm Uri.

Of the 1219 Texas nursing homes in 2021, 245 reported losing power or water during Winter Storm Uri. The steps for determining our final analytical sample are shown in eFigure 1 in Supplement 1. We excluded 45 nursing homes with no residents who stayed in the facility for at least 100 days (ie, long-stay residents) on January 1, 2021.^22^ Consistent with prior studies,^23,24,25^ we focused on long-stay nursing home residents because they may be more vulnerable to hospitalization or mortality than short-stay residents.^17^ Additionally, nursing homes have been shown to increase the percentage of short-stay residents discharged home before a natural disaster occurs.^26^

### Measures

The independent variable was whether a nursing home reported a loss of power or water, 2 serious consequences from a major weather event that can disrupt nursing home care.^27^ According to Texas Health and Human Services,^19^ nursing homes that lost power included those that reported experiencing blackouts or no electricity, and those that lost water included those that reported having no water, a water shortage, or a burst pipe. Nursing homes did not report how long an outage occurred or if they experienced multiple power or water outages. Thus, we dichotomized each exposure as experiencing a power outage (yes or no) or a water outage (yes or no). Our main analyses combined nursing homes that had a power outage or water outage. As a sensitivity analysis, we differentiated between nursing homes that only had a power outage, only had a water outage, or had both.

We obtained 4 outcomes for each long-stay resident in a week using Medicare data: mortality, any clinician visits in the nursing home, any ED visits, and any hospitalizations. We dichotomized the health care use outcomes because few residents had multiple clinician visits, ED visits, and hospitalizations in a single week. We identified whether a resident died in that week on the basis of the date of death from the beneficiary summary file. We defined clinician visits as any clinician evaluation and management visits in a nursing home using Medicare evaluation and management bill (*Current Procedural Terminology* codes 99304-99310 and 99315-99318).^28^ We used claims and encounters from inpatient, outpatient, and carrier to determine whether a resident had any ED visits.^22^ We used inpatient claims and encounters to identify any hospitalization visits.^29^

### Statistical Analysis

We defined the Winter Storm Uri as being from February 14, to February 17, 2021.^6^ Nursing homes with a loss of power or water were the exposure group, while facilities without such loss became the control group. We first compared facility and resident characteristics in the exposure group and the control group. We then examined additional changes in outcomes after Winter Storm Uri using a differences-in-differences (DiD) framework. Theoretically, the outcomes in Texas nursing homes with vs without power or water outages should be similar to before the storm; hence, additional changes in health care utilization and mortality after the storm can be attributed to the storm. To assess the parallel trends assumption, we used a 2-level logistic regression model that included a continuous measure for time (ie, the 6 weeks before Winter Storm Uri), an indicator variable for whether a nursing home experienced a power or water outage during the storm, and an interaction term for time by outage status. Because the *F* statistic for the interaction term was not statistically significant, we concluded that the parallel trend assumption was valid. The DiD models compared outcomes in the 2 to 5 weeks after Winter Storm Uri (February 28 to March 27, 2021) vs outcomes in the 4 weeks to 1 week before Winter Storm Uri (January 17 to February 13, 2021). We excluded the week of and 1 week after the storm to allow time to develop adverse events such as hospitalization and death. Separate 2-level (residents nested in nursing home facilities) logistic regression models estimated the additional changes in health care utilization and mortality due to losing power or water. These models included time (pre– vs post–Winter Storm Uri), outage (loss of power or water vs no loss), an interaction between time and outage, and residents’ age, sex, race and ethnicity, and Medicaid status. Finally, we described the weekly percentage of residents who died, and had any clinician visits, ED visits, or hospitalizations during the 6 weeks before (weeks −6 to −1), the week of (week 0), and 6 weeks after (weeks 1 to 6) Winter Storm Uri. For the mortality analysis, the denominator for each week included residents who were alive on the first day of that week. For the outcomes of clinician visits, ED visits, and hospitalizations, the denominators were residents who were alive on the first day of the week and were currently enrolled in Medicare Parts A and B. All analyses were performed using SAS Enterprise 7.1 (SAS Institute) at the CMS Virtual Research Data Center from March 2024 to January 2025. The threshold for statistical significance was a 2-sided *P* < .05.

## Results

### Long-Stay Resident and Nursing Home Characteristics

The sample included 45 439 long stay residents from 1174 nursing homes. There were 8521 long-stay residents in 231 facilities that reported outages (mean [SD] age, 80.07 [12.21] years; 5664 female [66.47%]; 1230 Black [13.43%]; 1449 Hispanic [17.01%]; 5702 non-Hispanic White [66.92%]) and 36 918 in 943 facilities that did not report outages (mean [SD] age, 80.42 [11.92] years; 12 705 female [65.59%]; 2081 Black [13.76%]; 6926 Hispanic [18.76%]; 24 122 non-Hispanic White [65.34%]). There were higher proportions of residents who were younger, Black, non-Hispanic White, and enrolled in Medicaid in nursing homes that reported losing power or water than homes that did not report losing power or water (eTable 1 in Supplement 1). Of 1174 Texas nursing homes, 231 (19.68%) reported power or water outages during Winter Storm Uri. There were no statistically significant differences in the bed size, location (urban or rural), or ownership of nursing homes that reported losing power or water compared with those that did not (eTable 2 in Supplement 1).

### Locations of Texas Nursing Homes That Lost Power or Water

**Figure 1.**
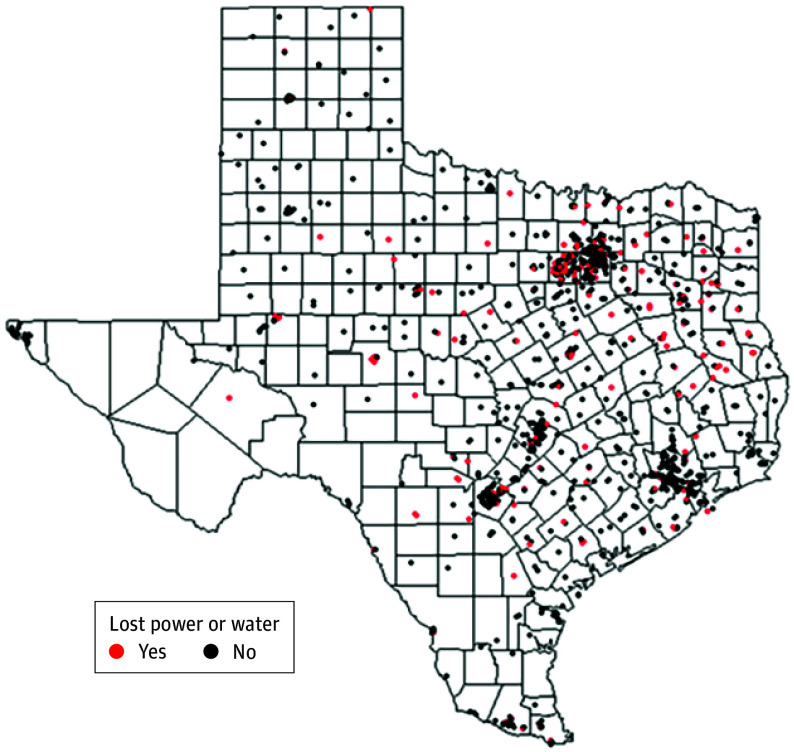
Texas Nursing Homes Reporting Power or Water Outages During Winter Storm Uri A total of 231 out of 1174 Texas nursing homes reported power or water outages during Winter Storm Uri.

Figure 1 shows the geocoded locations of all Texas nursing homes. The metropolitan statistical areas (MSA) with the most nursing homes were Dallas-Fort Worth (240 nursing homes), Houston (159 nursing homes), San Antonio-New Braunfels (96 nursing homes), and Austin (61 nursing homes). A total of 231 nursing homes in our sample reported losing power or water during Winter Storm Uri. The percentage of nursing homes that reported losing power or water was higher in the Dallas-Fort Worth (56 nursing homes [23.33%]) and San Antonio-New Braunfels (18 nursing homes [21.88%]) MSAs than the Houston (18 nursing homes [11.32%]) and Austin (6 nursing homes [9.83%]) MSAs.

### Mortality

**Figure 2.**
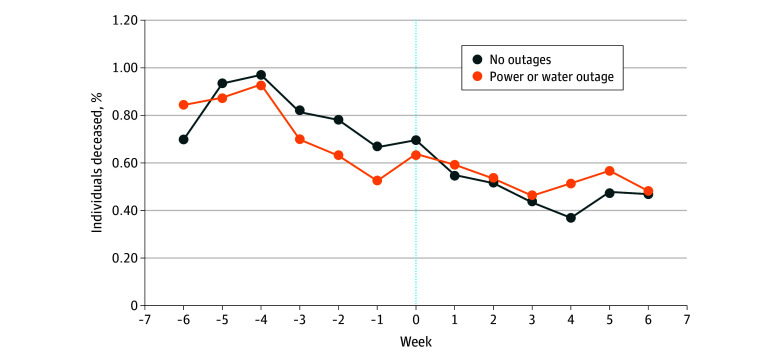
Weekly Mortality Rates Among Long-Stay Nursing Home Residents Before and After Winter Storm Uri The outage group included 8521 long-stay residents in 231 nursing homes that reported power or water outages, while the nonoutage group included 36 918 residents in 943 nursing homes without power or water outages. The outcome was the percentage of long-stay residents who died in a week. The denominator for each week included residents who were alive on the first day of each week. The week of the winter storm (week 0) was from February 13 to February 20, 2021.

Overall, weekly mortality rates decreased in the 6 weeks before Winter Storm Uri, which were similar to trends in other southern states like Georgia and Florida (eFigure 2 in Supplement 1). The findings from the DiD estimates indicated that loss of power or water was associated with a 0.13% (95% CI, 0.03%-0.23%) higher mortality rate (Table). Figure 2 shows that the mortality rates were higher in weeks 3 to 5 for nursing homes that reported losing power or water (37 of 8001 deceased [0.46%] to 45 of 7923 deceased [0.57%]) than those that did not (150 of 34 540 deceased [0.43%] to 164 of 34 263 deceased [0.48%]).

**Table.** Difference-in-Differences Examining Additional Changes in Mortality and Health Care Utilization Associated With Loss of Power or Water During Winter Storm Uri

Outcome	Differences-in-differences, % (95% CI)			*P* value	*P* for parallel trend
	Residents in nursing homes with power or water outages (post-Uri vs pre-Uri)	Residents in nursing homes without power and water outages (post-Uri vs pre-Uri)^a^	Difference-in-differences estimate associated with power or water outages^b^		
Mortality	−0.15 (−0.25 to −0.06)	−0.28 (−0.31 to −0.25)	0.13 (0.03 to 0.23)	.01	.26
Clinician visits	−1.82 (−2.52 to −1.11)	−1.51 (−1.83 to −1.20)	−0.31 (−1.08 to 0.47)	.44	.45
Emergency department visits	0.01 (−0.21 to 0.21)	−0.11 (−0.21 to −0.02)	0.11 (−0.12 to 0.34)	.33	.35
Hospitalizations	−0.03 (−0.18 to 0.12)	−0.10 (−0.17 to −0.03)	0.07 (−0.10 to 0.23)	.41	.51

^a^ The decreasing mortality rate in the control nursing homes was mainly due to the introduction of COVID-19 vaccines in early 2021.

^b^ The analysis compared outcomes from weeks 2 to 5 (post-Uri) with weeks −4 to −1 (pre-Uri). The week of the winter storm and the week right after were excluded. The difference-in-differences models used a 2-level (resident and facility) logistic regression with post-Uri, outage, post-Uri × outage, and resident demographic characteristics.

### Health Care Utilization

#### Clinician Visits

**Figure 3.**
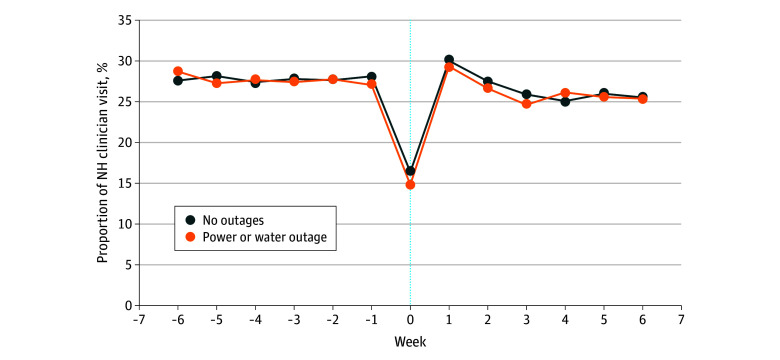
Weekly Percentage of Long-Stay Nursing Home (NH) Residents With Any Clinician Visit Before and After Winter Storm Uri The outage group included 8258 long-stay residents in 231 nursing homes that reported power or water outages, while the nonoutage group included 35 786 residents in 943 nursing homes without power or water outages. Both groups had Medicare inpatient and outpatient coverages. The outcome was the percentage of long-stay residents who received any clinician visit in the NH in a week. The week of the winter storm (week 0) was from February 13 to February 20, 2021.

We did not detect a statistically significant difference in clinician visits in the 4 weeks after the storm according to losing power or water (DiD, −0.31% 95% CI, −1.08% to 0.47%) (Table). For all nursing homes, approximately 15% of long-stay residents (6741 of 41 961 residents [16.10%]) had a clinician visit during the week of Winter Storm Uri. This percentage increased to 29.90% (12 471 of 41 667 residents) 1 week after the storm and then declined to 25.70% (10 581 of 41 216 residents) 3 weeks after the storm (Figure 3), similar to the percentage of residents who had a clinician visit before Winter Storm Uri.

#### ED Visits

**Figure 4.**
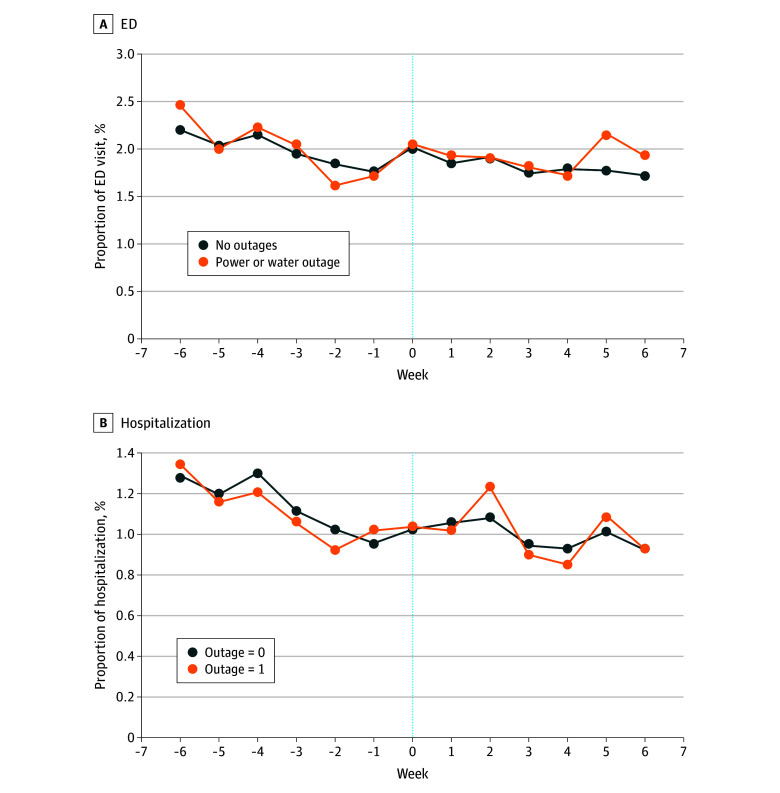
Weekly Percentage of Long-Stay Nursing Home Residents Who Had Any Emergency Department (ED) Visit or Hospitalization Before and After Winter Storm Uri The outage group included 8258 long-stay residents in 231 nursing homes that reported power or water outages, while the nonoutage group included 35 786 residents in 943 nursing homes without power and water outages. Both groups had Medicare inpatient and outpatient coverage. The outcome was the percentage of long-stay residents who had any ED visit or hospitalization in a week. The winter storm week (week 0) was from February 13 February 20, 2021.

Nursing homes that did not lose power or water showed a greater decrease in weekly ED rates after the storm (688 of 34 074 residents [2.02%] at week 0 vs 569 of 33 039 residents [1.72%] at week 6) than nursing homes that did lose power or water (162 of 7887 residents [2.05%] at week 0 vs 147 of 7631 residents [1.93] at week 6) (Figure 4A). However, the DiD estimates (Table) indicated that loss of power or water was not significantly associated with ED visits (DiD, 0.11%; 95% CI, −0.12% to 0.34%).

#### Hospitalizations

The weekly trend in the percentage of long-stay residents with any hospitalizations for nursing homes that reported losing power or water during Winter Storm Uri was similar to residents of nursing homes that did not lose power or water (Figure 4). Additionally, the DiD findings showed that loss of power or water was not associated with significant differences in hospitalizations (DiD, 0.07%; 95% CI, −0.10% to 0.23%).

### Sensitivity Analysis

Long-stay residents of nursing homes that lost power and water had significantly higher mortality than nursing homes that did not report power or water outages (DiD, 0.60%; 95% CI, 0.04%-1.16%), but differences in clinician visits, ED visits, and hospitalizations were not statistically significant (eTable 3 in Supplement 1). Additionally, the differences in mortality, clinician visits, ED visits, and hospitalizations after Winter Storm Uri were not statistically significant for long-stay residents of nursing homes that reported a power outage only (eTable 4 in Supplement 1) or water outage only compared with nursing homes that did not have any outages (eTable 5 in Supplement 1).

## Discussion

This cohort study investigated the association of power and water outages among nursing homes in Texas during Winter Storm Uri with the mortality and health care use of long-stay nursing home residents. We found that residents of nursing homes that reported a power or water outage had significantly higher mortality, but not hospitalizations or ED visits, in the 6 weeks after Winter Storm Uri than residents of nursing homes that did not report any outages. Although clinician visits to long-stay nursing home residents were nearly 50% lower during the week of Winter Storm Uri, clinician visits returned to prestorm levels 1 week after the storm and were not different between nursing homes that did and did not report a utility outage. We also did not detect differences in clinician visits according to whether a nursing home reported experiencing a power or water outage.

Prior studies on the association of utility outages with health outcomes of older adults have focused on power outages and community-dwelling older adult populations.^30,31,32^ An exception is a study of all nursing homes in Florida that found power outages were associated with increased 7-day and 30-day mortality for nursing home residents.^27^ We build on this study by investigating an unprecedented winter storm in Texas and considering multiple types of utility outages. Although power or water outages alone were not associated with residents’ mortality, nursing homes that reported both outages had significantly higher mortality. A loss of power during Winter Storm Uri would have put a nursing home in a challenging situation^19^ because many residents use medical devices that need a power supply and adequate lighting is important for keeping residents safe. A loss of water or boil water advisory could have put residents at risk because it would have become difficult for staff to maintain residents’ physical and oral hygiene,^33^ which is important for preventing infections that can increase mortality.^34,35^ While Winter Storm Uri disrupted the daily operations of nursing homes across Texas, facilities that had both types of outages likely experienced the greatest disruptions, which could have led to the higher mortality of long-stay residents in these facilities.

Texas is unprepared for major winter storms, and removing snow and ice from roads during Winter Storm Uri was a major issue.^36^ Consequently, hazardous driving conditions made traveling difficult or impossible for essential personnel,^36^ which is likely a major reason why we found that the percentage of long-stay nursing home residents who had a clinician visit sharply declined during the week of Winter Storm Uri, regardless of whether a nursing home reported experiencing a utility outage. This finding is consistent with evidence that daily staffing levels for registered nurses, certified nursing assistants, and licensed practical nurses decreased during the week of Winter Storm Uri, especially during the first 3 days of the storm.^37^

Texas is increasingly experiencing prolonged cold temperatures that strain its electrical grid as energy demands spike.^38^ This increase makes it important to continue investigating the association of utility outages during Winter Storm Uri with the health care outcomes of long-stay nursing home residents. First, thorough analyses of whether resident outcomes associated with utility outages varied according to nursing home characteristics, such as a facility’s overall 5-star rating, are warranted. A nursing home’s overall 1- to 5-star rating, which is meant to indicate the overall quality of a facility,^39^ is associated with better resident outcomes,^40^ including lower mortality, fewer hospitalizations, and fewer ED visits. High-rated nursing homes may be better able to prepare for an impending natural disaster (eg, increasing staffing) than low-rated facilities,^41^ which could contribute to fewer adverse resident outcomes. Second, the outcomes of nursing home residents should be compared with other states impacted by Winter Storm Uri. Texas is unique because it has minimal federal regulations overseeing its electrical infrastructure, and the electrical grid is separate from other states.^42^ Consequently, Texas cannot rely on other states for electricity in emergent situations when energy demands are high.

### Limitations

This study has limitations. Nursing homes voluntarily reported the power and water outages during Winter Storm Uri, and it is plausible that not all nursing homes that experienced a power outage or loss of water reported such events. The possible misclassification of nursing homes may have underestimated the actual effect size of the association of power and water outages during Winter Storm Uri with the mortality and health care utilization of Texas nursing home residents. Additionally, nursing homes were not asked to report how long they were without power or water, which prevents us from assessing the dose-response association of outages with adverse outcomes. Our analyses also did not include measures of the COVID-19 pandemic or influenza,^43^ which were ongoing during Winter Storm Uri.

## Conclusions

Our findings provide further evidence that nursing homes need access to resources that can keep residents safe during a disaster event and in the weeks that follow. Our findings also highlight the need for climate change adaptation efforts and improving nursing homes’ preparedness for such events.^44^ Winter Storm Uri brought to light the vulnerability and lack of preparedness of Texas infrastructure for natural disasters. A concern is the increasingly warm summers in Texas, and recurring major weather events will make prolonged power outages a more common experience. For the US to protect some of its most vulnerable populations in the era of climate change, including those that live in nursing homes, more research and policy solutions are needed and should be prioritized at the local, state, and federal levels.

## Notes

Supplement 1. **eFigure 1.** Cohort Selection **eTable 1.** Characteristics of Long-Stay Nursing Home Residents in Texas According to the Nursing Home Reporting a Power or Water Outage During Winter Storm Uri **eTable 2.** Characteristics of Nursing Homes in Texas According to Reporting a Power or Water Outage During Winter Storm Uri **eFigure 2.** Weekly Mortality Rates Among Long-Stay Nursing Home Residents in Georgia and Florida Before and After Winter Storm Uri **eTable 3.** Difference-in-Differences Examining Additional Changes in Mortality and Healthcare Utilization Associated With Loss of Power and Water During Winter Storm Uri **eTable 4.** Difference-in-Differences Examining Additional Changes in Mortality and Healthcare Utilization Associated With Loss of Power Only During Winter Storm Uri **eTable 5.** Difference-in-Differences Examining Additional Changes in Mortality and Healthcare Utilization Associated With Loss of Water Only During Winter Storm Uri

Supplement 2.
Data Sharing Statement
