# Supplementary material for: Winter Storm Uri, Mortality, and Health Care Use of Nursing Home Residents
Source: JAMA Netw Open. 2025 Apr 8;8(4):e254111. doi: 10.1001/jamanetworkopen.2025.4111 (PMC11979729; doi:10.1001/jamanetworkopen.2025.4111)
Supplement: Supplement 1. — eFigure 1. Cohort Selection eTable 1. Characteristics of Long-Stay Nursing Home Residents in Texas According to the Nursing Home Reporting a Power or Water Outage During Winter Storm Uri eTable 2. Characteristics of Nursing Homes in Texas According to Reporting a Power or Water Outage During Winter Storm Uri eFigure 2. Weekly Mortality Rates Among Long-Stay Nursing Home Residents in Georgia and Florida Before and After Winter Storm Uri eTable 3. Difference-in-Differences Examining Additional Changes in Mortality and Healthcare Utilization Associated With Loss of Power and Water During Winter Storm Uri eTable 4. Difference-in-Differences Examining Additional Changes in Mortality and Healthcare Utilization Associated With Loss of Power Only During Winter Storm Uri eTable 5. Difference-in-Differences Examining Additional Changes in Mortality and Healthcare Utilization Associated With Loss of Water Only During Winter Storm Uri [file jamanetwopen-e254111-s001.pdf]

Supplemental Online Content

Downer B, Holland A, Li S, Xu H. Winter Storm Uri and mortality and health care use of nursing home residents. *JAMA Netw. Open*. 2025;8(4):e254111.
doi:10.1001/jamanetworkopen.2025.4111

eFigure 1. Cohort Selection

eTable 1. Characteristics of Long-Stay Nursing Home Residents in Texas According to the Nursing Home Reporting a Power or Water Outage During Winter Storm Uri

eTable 2. Characteristics of Nursing Homes in Texas According to Reporting a Power or Water Outage During Winter Storm Uri

eFigure 2. Weekly Mortality Rates Among Long-Stay Nursing Home Residents in Georgia and Florida Before and After Winter Storm Uri

eTable 3. Difference-in-Differences Examining Additional Changes in Mortality and Healthcare Utilization Associated With Loss of Power and Water During Winter Storm Uri

eTable 4. Difference-in-Differences Examining Additional Changes in Mortality and Healthcare Utilization Associated With Loss of Power Only During Winter Storm Uri

eTable 5. Difference-in-Differences Examining Additional Changes in Mortality and Healthcare Utilization Associated With Loss of Water Only During Winter Storm Uri

This supplemental material has been provided by the authors to give readers additional information about their work.

eFigure 1: Cohort selection

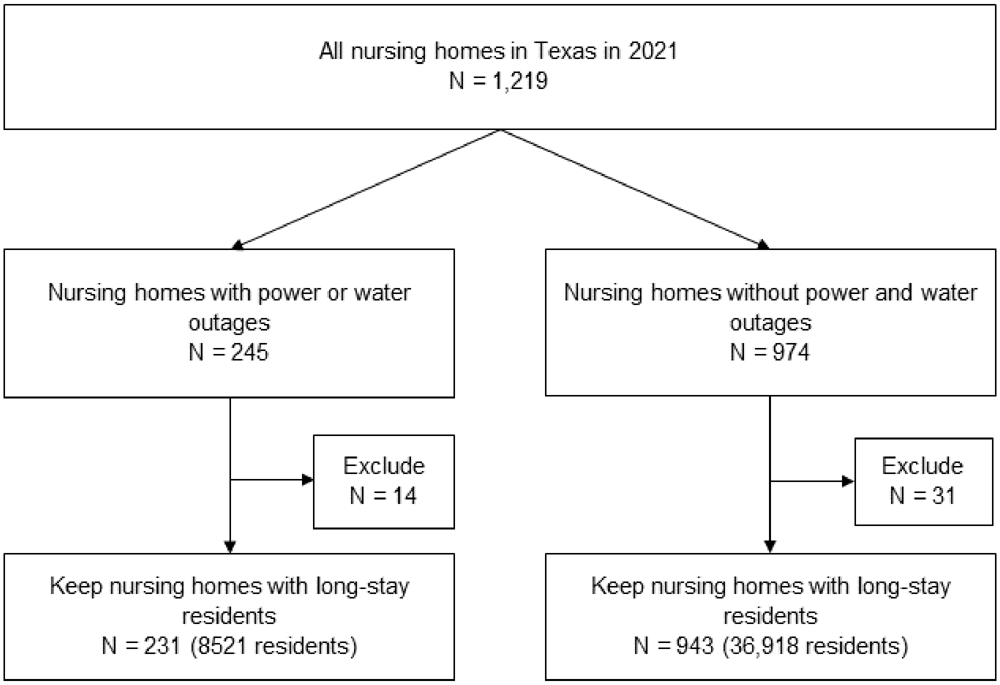

Long-stay residents included residents who stayed in the nursing home for at least 100 days on 1/1/2021.

© 2025 Downer B et al. *JAMA Netw. Open*.

eTable 1: Characteristics of long-stay nursing home residents in Texas according to the nursing home reporting a power or water outage during Winter Storm Uri.

Resident characteristics	Residents in nursing homes with power outages or water shortages (N=8,521)	Residents in nursing homes without power or water outage (N=36,918)	P value
	N (%)	N (%)	
Age, mean (SD)	80.07 (12.21)	80.42 (11.92)	0.02
Gender			0.12
Male	2,857 (33.53%)	12,705 (34.41%)	
Female	5,664 (66.47%)	24,213 (65.59%)	
Race and ethnicity			<0.001
Non-Hispanic White	5,702 (66.92%)	24,122 (65.34%)	
Black	1,230 (14.43%)	2,081 (13.76%)	
Hispanic	1,449 (17.01%)	6,926 (18.76%)	
Other	140 (1.64%)	789 (2.14%)	
Medicaid coverage			<0.001
No	3,978 (46.68%)	18,046 (48.88%)	
Yes	4,543 (53.32%)	18,872 (51.12%)	

Note: Long-stay residents included residents who stayed in the nursing home for at least 100 days on 1/1/2021.

© 2025 Downer B et al. *JAMA Netw. Open*.

eTable 2: Characteristics of nursing homes in Texas according to reporting a power or water outage during Winter Storm Uri.

**Facility characteristics**	**Nursing homes with power or water outages (N=231)**	**Nursing homes without power or water outages (N=943)**	**P value**
	**N (%)**	**N (%)**	
Bed size, Mean (SD)	114.88 (37.77)	115.54 (38.28)	0.82
Location			0.53
Rural	69 (29.87%)	262 (27.78%)	
Urban	162 (70.13%)	681 (72.22%)	
Ownership			0.57
Non-profit	15 (6.49%)	65 (6.89%)	
Government	26 (11.26%)	130 (13.79%)	
Profit	190 (82.25%)	748 (79.32%)	

© 2025 Downer B et al. *JAMA Netw. Open*.

eFigure 2: Weekly mortality rates among long-stay nursing home residents in Georgia and Florida before and after Winter Storm Uri.

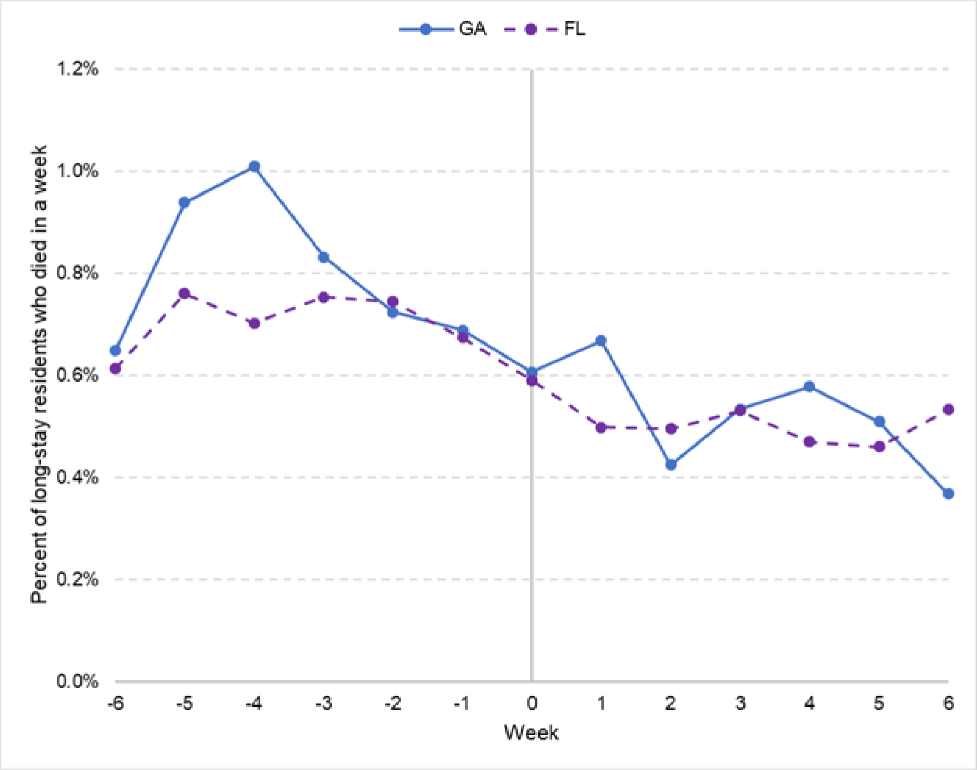

The analysis included 1,8029 long-stay residents in 353 Georgia nursing homes, 35,380 long-stay residents in 677 Florida nursing homes The outcome was the percentage of long-stay residents who died in a week. The denominator for each week included residents who were alive on the first day of each week. The week of the Texas winter storm (week 0) was from 2/13/2021 to 2/20/2021.

© 2025 Downer B et al. *JAMA Netw. Open*.

eTable 3: Difference-in-differences examining additional changes in mortality and healthcare utilization associated with loss of power and water during Winter Storm Uri

Outcome	Residents in nursing homes with power and water outages (Post-pre)	Residents in nursing homes without power or water outages (Post-pre)[a]	Difference-in-differences estimate associated with power and water outages[b]	P value	P-value for parallel trend
Mortality rate	0.31% (-0.24%, 0.87%)	-0.29% (-0.32%, -0.25%)	0.60% (0.04%, 1.16%)	0.04	0.18
Hospitalization rate	-0.06% (-0.76%, 0.65%)	-0.10% (-0.17%, -0.03%)	0.04% (-0.67%, 0.75%)	0.91	0.98
Rate of emergency department visit	-0.25% (-1.24%, 0.75%)	-0.11% (-0.21%, -0.02%)	-0.14% (-1.14 %, 0.86%)	0.79	0.69
Any clinician visit	-1.33% (-4.62%, 1.97%)	-1.51% (-1.83%, -1.20%)	0.18% (-3.13%, 3.49%)	0.91	0.07

Notes: [a] The decreasing mortality rate in the control nursing homes was mainly driven by the introduction of COVID-19 vaccines in early 2021.

[b] The analysis compared outcomes from weeks 2 to 5 (post) to weeks -4 to -1 (pre). The week of the winter storm and the week right after were excluded. The Difference-in-differences models used a two-level (resident, facility) logistic regression with post, outage, post*outage, and resident demographic characteristics.

© 2025 Downer B et al. *JAMA Netw. Open*.

eTable 4: Difference-in-differences examining additional changes in mortality and healthcare utilization associated with loss of power only during Winter Storm Uri

Outcome	Residents in nursing homes with power outages only (Post-pre)	Residents in nursing homes without power or water outages (Post-pre)[a]	Difference-in-differences estimate associated with power outages only [b]	P value	P-value for parallel trend
Mortality rate	-0.16% (-0.31%, -0.01%)	-0.28% (-0.32%, -0.25%)	0.12% (-0.03%, 0.27%)	0.12	0.05
Hospitalization rate	0.05% (-0.15%, 0.26%)	-0.10% (-0.17%, -0.03%)	0.15% (-0.07%, 0.37%)	0.17	0.38
Rate of emergency department visit	0.09% (-0.19%, 0.37%)	-0.11% (-0.21%, -0.02%)	0.20% (-0.09%, 0.50%)	0.17	0.73
Any clinician visit	-2.02% (-2.98%, -1.06%)	-1.51% (-1.83%, -1.20%)	-0.51% (-1.52%, 0.51%)	0.33	0.09

Notes: [a] The decreasing mortality rate in the control nursing homes was mainly driven by the introduction of COVID-19 vaccines in early 2021.

[b] The analysis compared outcomes from weeks 2 to 5 (post) to weeks -4 to -1 (pre). The week of the winter storm and the week right after were excluded. The Difference-in-differences models used a two-level (resident, facility) logistic regression with post, outage, post*outage, and resident demographic characteristics.

© 2025 Downer B et al. *JAMA Netw. Open*.

eTable 5: Difference-in-differences examining additional changes in mortality and healthcare utilization associated with loss of water only during Winter Storm Uri

Outcome	Residents in nursing homes with water outages only (Post-pre)	Residents in nursing homes without power or water outages (Post-pre)[a]	Difference-in-differences estimate associated with water outages only[b]	P value	P-value for parallel trend
Mortality rate	-0.17% (-0.32%, -0.02%)	-0.29% (-0.32%, -0.25%)	0.12% (-0.04%, 0.27%)	0.14	0.20
Hospitalization rate	-0.13% (-0.36%, 0.10%)	-0.10% (-0.17%, -0.03%)	-0.03% (-0.27%, 0.21%)	0.80	0.91
Rate of emergency department visit	-0.08% (-0.41%, 0.24%)	-0.11% (-0.21%, -0.01%)	0.03% (-0.31%, 0.37%)	0.87	0.33
Any clinician visit	-1.80% (-2.99%, -0.62%)	-1.69% (-2.04%, -1.33%)	-0.12% (-1.36%, 1.13%)	0.85	0.45

Notes: [a] The decreasing mortality rate in the control nursing homes was mainly driven by the introduction of COVID-19 vaccines in early 2021.

[b] The analysis compared outcomes from weeks 2 to 5 (post) to weeks -4 to -1 (pre). The week of the winter storm and the week right after were excluded. The Difference-in-differences models used a two-level (resident, facility) logistic regression with post, outage, post*outage, and resident demographic characteristics.

© 2025 Downer B et al. *JAMA Netw. Open*.
