# Supplementary material for: Winter Storm Uri, Mortality, and Health Care Use of Nursing Home Residents
Source: JAMA Netw Open. 2025 Apr 8;8(4):e254111. doi: 10.1001/jamanetworkopen.2025.4111 (PMC11979729; doi:10.1001/jamanetworkopen.2025.4111)
Supplement: Supplement 2. — Data Sharing Statement [file jamanetwopen-e254111-s002.pdf]

Data Sharing Statement

Downer. Winter Storm Uri and Mortality and Health Care Use of Nursing Home Residents. *JAMA Netw Open*. Published April 08, 2025. doi:10.1001/jamanetworkopen.2025.4111

Data
Data available: No

Additional Information
Explanation for why data not available: Our study included the claims files for Medicare beneficiaries in Texas. Access to these data is restricted to users with an approved Data Use Agreement with CMS.
